# Gene expression in extratumoral microenvironment predicts clinical outcome in breast cancer patients

**DOI:** 10.1186/bcr3152

**Published:** 2012-03-19

**Authors:** Erick Román-Pérez, Patricia Casbas-Hernández, Jason R Pirone, Jessica Rein, Lisa A Carey, Ronald A Lubet, Sendurai A Mani, Keith D Amos, Melissa A Troester

**Affiliations:** 1 Department of Epidemiology, University of North Carolina at Chapel Hill, Campus Box 7435, Chapel Hill, NC 27599, USA; 2 Department of Pathology and Laboratory Medicine, University of North Carolina at Chapel Hill, Campus Box 7525, Chapel Hill, NC 27599, USA; 3 Lineberger Comprehensive Cancer Center, University of North Carolina at Chapel Hill, Campus Box 7295, Chapel Hill, NC 27599, USA; 4 Division of Cancer Prevention, National Cancer Institute, 6130 Executive Blvd, Bethesda, MD 20892 USA; 5 Department of Molecular Pathology, University of Texas, Houston, TX 77053, USA; 6 Metastasis Research Center, M. D. Anderson Cancer Center, University of Texas, Unit Number 951, Houston, TX 77053, USA; 7 UNC Department of Surgery, University of North Carolina at Chapel Hill, Campus Box 7050, Chapel Hill, NC 27599, USA

## Abstract

**Introduction:**

A gene expression signature indicative of activated wound responses is common to more than 90% of non-neoplastic tissues adjacent to breast cancer, but these tissues also exhibit substantial heterogeneity. We hypothesized that gene expression subtypes of breast cancer microenvironment can be defined and that these microenvironment subtypes have clinical relevance.

**Methods:**

Gene expression was evaluated in 72 patient-derived breast tissue samples adjacent to invasive breast cancer or ductal carcinoma *in situ*. Unsupervised clustering identified two distinct gene expression subgroups that differed in expression of genes involved in activation of fibrosis, cellular movement, cell adhesion and cell-cell contact. We evaluated the prognostic relevance of extratumoral subtype (comparing the Active group, defined by high expression of fibrosis and cellular movement genes, to the Inactive group, defined by high expression of claudins and other cellular adhesion and cell-cell contact genes) using clinical data. To establish the biological characteristics of these subtypes, gene expression profiles were compared against published and novel tumor and tumor stroma-derived signatures (Twist-related protein 1 (TWIST1) overexpression, transforming growth factor beta (TGF-β)-induced fibroblast activation, breast fibrosis, claudin-low tumor subtype and estrogen response). Histological and immunohistochemical analyses of tissues representing each microenvironment subtype were performed to evaluate protein expression and compositional differences between microenvironment subtypes.

**Results:**

Extratumoral Active versus Inactive subtypes were not significantly associated with overall survival among all patients (hazard ratio (HR) = 1.4, 95% CI 0.6 to 2.8, *P *= 0.337), but there was a strong association with overall survival among estrogen receptor (ER) positive patients (HR = 2.5, 95% CI 0.9 to 6.7, *P *= 0.062) and hormone-treated patients (HR = 2.6, 95% CI 1.0 to 7.0, *P *= 0.045). The Active subtype of breast microenvironment is correlated with TWIST-overexpression signatures and shares features of claudin-low breast cancers. The Active subtype was also associated with expression of TGF-β induced fibroblast activation signatures, but there was no significant association between Active/Inactive microenvironment and desmoid type fibrosis or estrogen response gene expression signatures. Consistent with the RNA expression profiles, Active cancer-adjacent tissues exhibited higher density of TWIST nuclear staining, predominantly in epithelium, and no evidence of increased fibrosis.

**Conclusions:**

These results document the presence of two distinct subtypes of microenvironment, with Active versus Inactive cancer-adjacent extratumoral microenvironment influencing the aggressiveness and outcome of ER-positive human breast cancers.

## Introduction

Gene expression analysis of tissue adjacent to invasive breast cancer and ductal carcinoma *in situ *has suggested that intratumoral stromal responses contribute to disease progression. Finak *et al. *[1] showed that elevated expression of stroma-derived immune mediators in tumor tissue predicted relapse. Chang *et al. *reported a signature of fibroblast response [2] and Beck *et al. *reported fibromatosis and macrophage-associated signatures, each with prognostic value [3,4]. Stromal responses are activated at early stages in carcinogenesis, even in the absence of invasion [5], leading to speculation that "for acquisition of the invasive phenotype, the stroma is dominant over the epithelium" [6]. We recently reported an *in vivo *wound response signature derived from tissue adjacent to breast cancer, which when expressed in tumors, predicts relapse and overall survival [7]. The vast majority of studies evaluating stroma-derived signatures [1-5,8-11] have focused on intratumoral stromal expression rather than extratumoral expression.

Growing evidence suggests that extratumoral microenvironment may play a role in cancer progression. Chen *et al. *showed that some cancer patients have gene expression patterns in their adjacent non-neoplastic tissue that are similar to invasive breast cancer signatures, and that these signatures may predict progression of early premalignant lesions [12]. Graham *et al. *also found that gene expression in normal epithelium of ER positive and ER negative breast cancers echoes the ER status of the adjacent tumors [13]. These observations suggest that the stroma and/or epithelium adjacent to tumors may harbor changes, referred to as field effects [14], and that these changes may be of prognostic value. However, an investigation of genomic heterogeneity in the extratumoral microenvironment, independent of effects caused by adjacent tumor characteristics, has not been reported. Identification of gene expression subtypes in the extratumoral microenvironment may provide important insights into how stromal response alters the progression of disease.

To evaluate the hypothesis that extratumoral microenvironment influences disease progression, we used cancer-adjacent non-neoplastic tissue from 72 invasive breast cancer and ductal carcinoma *in situ *cases to identify distinct gene expression subtypes in extratumoral tissue. Biological features of these subtypes were defined by comparison with established and novel gene expression signatures and by performing histological and immunohistochemical analyses. The novel microenvironment subtypes identified were also evaluated for associations with overall survival. Our data suggest two biologically distinct subtypes of extratumoral microenvironment with distinct biological features and clinical outcomes.

## Materials and methods

### Patient samples

Patients were women undergoing mastectomy at University of North Carolina Hospitals in Chapel Hill, NC. All patients enrolled voluntarily under Institutional Review Board-approved protocols. For histologically normal tissue adjacent to breast cancer or ductal carcinoma *in situ*, a pathologist from the Lineberger Comprehensive Cancer Center's Tissue Procurement Facility at the University of North Carolina in Chapel Hill confirmed that a mirror specimen adjacent to that used for RNA isolation was histologically benign.

Patients with invasive cancers (*n *= 68) and ductal carcinoma *in situ *(DCIS) (*n *= 4) were considered. Patients receiving neoadjuvant therapy were excluded. ER-positive patients were identified by pathologic report and endocrine-treated patients were defined as cancer patients whose medical records reflect that they received anti-estrogen therapy, such as tamoxifen or aromatase inhibitors. Median follow-up time for the 72 patients was 39 months. All tissues were handled by snap freezing immediately after surgery. RNA was isolated using established protocols as described previously [7].

To evaluate intra-individual variation in the signatures we identified, we used duplicate specimen samples from the same individuals. For these analyses, biospecimens included five pairs of cancer-adjacent tissue pairs from the University of North Carolina Hospitals in Chapel Hill, NC and five pairs from a study conducted by the National Cancer Institute in Warsaw and Lodz, Poland [15]. For all 10 pairs, both peritumoral (< 2 cm) and remote (2+ cm) tissues were sampled.

### Cell lines and reagents for generating TWIST and TGFβ signatures

To identify a gene expression profile associated with TWIST overexpression, the non-tumorigenic immortalized human mammary epithelial cells (HMLE) and the HMLE cells stably expressing the transcription factor TWIST1 (HMLE-TWIST) [16] were cultured in HuMEC media with HuMEC Supplement and Bovine Pituitary Extract (GIBCO, Carlsbad CA, USA). HMLE-TWIST cells were harvested after 48 h in culture (at 80% confluence) and compared with HMLE cells harvested under an identical protocol. To identify a signature of fibroblast activation by transforming growth factor beta (TGF-β), hTERT-immortalized fibroblasts from reduction mammoplasty patients (RMF) [17] (maintained as described previously [18]), were treated with 50 pg TGF-β1 (PeproTech, Rockyhill, NJ, USA) for 48 h. TGF-β1 was reconstituted in 10 mM Citric Acid and 2 mg/mL albumin in PBS, with supplemented media replaced every 24 h. Gene expression profiles of TGFβ-treated RMF cells were compared to those in untreated RMFs.

### Microarray analysis

Microarrays were two-color Agilent 4X44k G4112F arrays (Agilent, Santa Clara, CA, USA) or custom 244 k human arrays (Agilent G4502A). The probe set common to both platforms was used for all analyses as previously described [7]. Cy3-labeled reference was produced from total RNA from Stratagene Universal Human Reference (spiked with 1:1,000 with MCF-7 RNA and 1:1,000 with ME16C RNA to increase expression of breast cancer genes) following amplification with Agilent Quick Amp labeling kit following the manufacturer's protocol with minor modifications as described in Hu *et al. *[19]. The identical protocol was applied to Cy5-label total RNA from cell lines and cancer-adjacent tissue. Data are publicly available through the Gene Expression Omnibus (GSE31589).

### Unsupervised clustering

All data were loaded to the UNC Microarray Database for normalization and filtering. Only probes with signal intensity greater than 10 dpi in both channels were included. Data were lowess normalized, probes with at least 80% good data were selected, and missing data were imputed using k-nearest neighbors imputation with k = 10. To identify subtypes of microenvironment, we selected probes with an inter-quartile range (IQR) of at least 0.8. This probe set was used to perform unsupervised clustering of cancer-adjacent tissues (Cluster 3.0). Functional analysis of gene clusters in the unsupervised cluster was performed to identify significant functions with *P*-values less than 0.05 (using Ingenuity Pathway Analysis (IPA), with Benjamini-Hochberg multiple testing correction).

### Associations between extratumoral microenvironment subtypes and biologically-defined gene expression signatures

To characterize the biological phenotypes of the extratumoral subtypes, gene expression in each sample was compared to existing and novel gene expression signatures. TWIST1-related signatures reflecting cellular de-differentiation and activated stroma were identified via two-class unpaired Significance Analysis of Microarrays (SAM) [20] with a false discovery rate (FDR) < 0.01 comparing HMLE-TWIST with parental HMLE cell line (vector only) (Cell lines used are described in [21]). TGF-β1-dependent responses of fibroblasts were identified using two-class unpaired SAM with FDR < 0.01 comparing treated versus sham RMFs (as described above). The median centered expression profile of each individual patient was then evaluated for correlation with these signatures by calculating Pearson correlation coefficients, using the method of Creighton *et al. *[22]. Three published signatures, for desmoid-type fibrosis (DTF) [4], Claudin-low breast cancer [23] and estrogen response [24], were assessed the same way. Briefly, vectors corresponding to all genes in each of the five signatures were constructed, with 1 assigned to up-regulated genes and -1 assigned to down-regulated genes. A Pearson correlation coefficient was calculated for this standard vector versus the vector of median centered gene expression for each patient. Patients were classified as positive for a given signature if the Pearson correlation coefficient was greater than zero, and negative if the coefficient was less than zero.

After evaluating the 72 patient sample set as described, additional samples were microarrayed to evaluate prevalence of Active subtype as a function of distance to tumor. For each of 10 patients, 2 samples (one peritumoral and one remote) were analyzed by microarray and the 20-sample set was median-centered. Each sample was classified as Active or Inactive using the Pearson correlation coefficient for median-centered expression of that sample versus the vector of up (1) and down (-1) genes in the Active patients from the 72 patient set. Patients with a positive Pearson correlation coefficient were classified as Active (others were Inactive).

### Survival analysis

Associations between cluster group (Active versus Inactive) and overall survival were evaluated using Kaplan-Meier and Cox-Proportional Hazards analyses in R 2.11.0 (survival package). Univariate Cox models were used to estimate the hazard ratio (HR) and 95% CI. Hazard Ratios did not change substantially with exclusion of the one DCIS patient in this dataset. Relapse-free survival data were incomplete for a large number of patients and were underpowered in this dataset, but survival trends similar to those for overall survival were observed (data not shown).

### Immunohistochemistry

Formalin fixed mirror samples of non-neoplastic tissue adjacent to breast cancer were paraffin embedded and assayed by IHC to compare markers across the 72 Active and Inactive patients and in 5 pairs of peritumoral and remote specimens collected at UNC (where paraffin was available). Five micrometer paraffin sections were obtained, deparaffinized, hydrated, and antigen retrieval (HIER) was performed using Dako Target Retrieval Solution S1699(Dako, Glostrup Denmark). Sections were washed in 0.05 M Tris buffer pH 7.6 and treated with 3.0% hydrogen peroxide in dH_2_0 for 10 minutes to reduced endogenous peroxidase. Sections were blocked in Dako protein serum free solution (X0909) for 30 minutes and incubated overnight at 4°C with antisera against TWIST1 (Abcam, ab50887, Cambridge, UK)) diluted 1:200 in Dako antibody diluent (S0809). TWIST1 staining was revealed through the use of a biotinylated secondary (Jackson ImmunoResearch 115-065-166, Reston VA, USA), ABC complex (Standard Elite Vector Laboratories, PK6100, Burlingame, CA, USA) and reacted in diaminobenzidine (Invitrogen DAB Substrate Kit 00-2014, Carlbad, CA, USA). Subsequent hematoxylin or Masson's trichrome counterstaining was performed, slides were dehydrated, cleared in xylene and DPX (BDH 360294H) and cover-slipped for light microscopy at 10X. All slides were scanned using Aperio ScanScope CS and analyzed with Aperio Image Scope V11.0.2.725 (Vista, CA, USA) using the nuclear analysis algorithm Nuclear V9. TWIST-positive (2+ or 3+) nuclear counts per unit area of epithelium and per unit area of stroma were calculated.

## Results

### Identification of distinct extratumoral microenvironment subtypes adjacent to breast cancer

**Figure 1:**
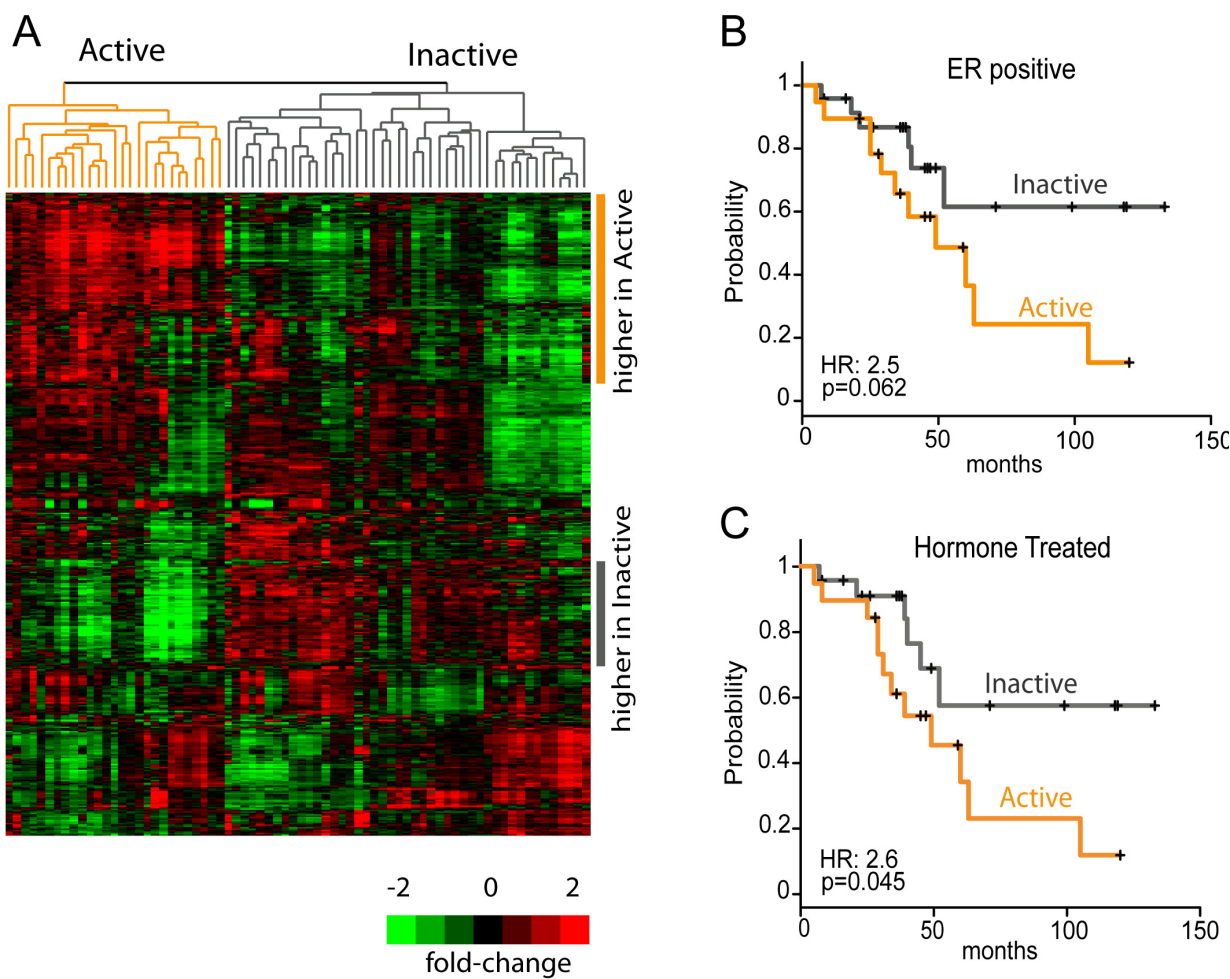
**Identification of extratumoral microenvironment subtypes adjacent to breast cancer**. **(A) **Unsupervised clustering dendrogram for 3,518 probes yields two main clusters of non-neoplastic tissues. Cluster 1/Active subtype (left, orange) is associated with high expression of genes involved in cellular movement, inflammatory response, fibrosis and low expression of genes involved in cellular adhesion and differentiation (See also Table 2 for gene ontology analyses). Cluster 2/Inactive Subtype has higher cellular adhesion and differentiation-related gene expression. Kaplan-Meier analysis and univariate Cox Proportional Hazards analysis (to estimate hazard ratios) were conducted comparing overall survival for Active versus Inactive. Results are presented for ER positive patients only **(B) **and for endocrine-treated patients **(C)**.

To identify gene expression subtypes of extratumoral microenvironment, we analyzed microarray data on 72 extratumoral tissues from women with invasive breast cancer or ductal carcinoma *in situ*. Unsupervised clustering on approximately 3,500 of the most variable probes resulted in two distinct clusters (Figure 1A, with complete gene list presented in Additional file 1: Table S1). Clusters were defined as 'Active' and 'Inactive' as described in Methods. Kaplan-Meier analysis for overall survival across all patients showed that patients with an Active signature in their extratumoral microenvironment had poorer overall survival (HR = 1.4, 95% CI: 0.6 to 2.8, *P *= 0.337), but the association was weak and not statistically significant when considering all breast cancer patients. However, extratumoral microenvironment is unlikely to show dominance over highly aggressive tumor biology and even tumor-derived signatures have poor accuracy in predicting ER-negative patient survival due to the uniformly poor survival of this group [25]. Therefore, we also assessed prognostic value of Active versus Inactive subtype among ER-positive (*n *= 43) and endocrine-treated (*n *= 42) patients. Among these patients, there was a strong association between Active subtype and overall survival (Figure 1B, C; ER-positive patients Active versus Inactive HR = 2.5, 95% CI: 0.9 to 6.7, *P *= 0.062; endocrine-treated patients Active versus Inactive HR = 2.6, 95% CI: 1.0 to 7.0, *P *= 0.045). These results suggest that the phenotype of the extratumoral microenvironment may have value as an independent predictor of ER-positive/hormone-treated patient outcome.

To further evaluate clinical implications of extratumoral subtypes, we analyzed the association of common clinical parameters with Active versus Inactive subtype. We found no statistically significant association between Active/Inactive subtype and standard prognostic clinico-pathological parameters, including breast cancer subtype, ER status, tumor size, and tumor grade (Table 1). Thus, the extratumoral microenvironment subtypes are independent of tumor subtype and do not appear to be strongly driven by the biology of the tumor. We also evaluated several clinical factors as potential confounders of the relationship between Active/Inactive subtype and survival. While this dataset is not large enough for well-powered multivariable analyses, the HR comparing Active to Inactive remained stable and above 2.0 even after adjusting for grade (high and medium versus low as two indicator variables), subtype (normal-like, luminal B, HER2 and basal-like versus luminal A as four indicator variables), size (< 2 cm versus >/ = 2 cm) and age (continuous).

**Table 1:** Clinical characteristics of 72 patients evaluated for extratumoral gene expression subtypes

	Total	Active, N (%)	Inactive, N (%)	
Menopausal Status				
Pre	32	10 (31.3)	22 (68.7)	
Post	32	15 (46.9)	17 (53.1)	
Missing	8	2 (25.0)	6 (75.0)	
Breast Cancer Subtype				
Normal-like	4	1 (25.0)	3 (75.0)	
Luminal A	22	8 (36.4)	14 (63.6)	
Luminal B	9	3 (33.3)	6 (66.7)	
Her2+	7	3 (42.9)	4 (57.1)	
Basal-like	12	4 (33.3)	8 (66.7)	
Missing	18	8 (44.4)	10 (55.6)	
ER status				
Positive	44	19 (43.2)	25 (56.8)	
Negative	23	7 (30.4)	16 (69.6)	
Missing	5	1 (20.0)	4 (80.0)	
HER2 status				
Positive	13	5 (38.5)	8 (61.5)	
Negative	38	12 (31.6)	26 (68.4)	
Missing	21	10 (47.6)	11 (52.4)	
Tumor size (cm)				
0 to 2	25	14 (56.0)	11 (44.0)	
2.1 to 4	24	9 (37.5)	15 (62.5)	
4+	19	4 (21.1)	15 (78.9)	
Missing	4	0 (0.0)	4 (100)	
Tumor grade				
1	6	3 (50.0)	3 (50.0)	
2	18	4 (22.2)	14 (77.8)	
3	28	13 (46.4)	15 (53.6)	
Missing	20	7 (35.0)	13 (65.0)	

### Biological relevance of microenvironment subtypes

To establish the biological character of the two clusters in Figure 1, we performed ontology analysis using Ingenuity Pathway Analysis (IPA) (Table 2). Genes highly expressed in the Active group (Figure 1A, upper right panel), were genes related to movement of cells (*P *= 3.94E-17), inflammatory response (*P *= 3.09E-15), connective tissue disorder (*P *= 6.22E-15), fibrosis (*P *= 2.63e-07), chemotaxis of cells (*P *= 6.23E-07) and recruitment of macrophages (*P *= 4.24E-04). Genes that were expressed at lower levels in the Active groups (Figure 1A, lower right panel) over-represented functional categories, such as adhesion of cells (*P *= 3.38E-06), differentiation of epithelial cells (*P *= 1.08E-03), formation of cell-cell contacts (*P *= 1.41E-03). Additional file 2: Table S2 shows full ontology analyses for the gene clusters highlighted in Figure 1 (IPA results).

**Table 2:** Pathway analysis of enriched ontological categories, major gene clusters demarcated in Figure 1

Category	Function annotation	Adjusted *P*-value^a^
**Highly expressed in Active**		
Cellular Movement	Movement of cells	3.94E-17
	Homing of cells	3.17E-07
	Chemotaxis of cells	6.23E-07
	Infiltration of cells	4.22E-05
Inflammatory Response	Inflammatory response	3.09E-15
	Infiltration by neutrophils	1.91E-04
	Recruitment of macrophages	4.24E-04
Connective Tissue Disorders	Connective tissue disorder	6.22E-15
Organismal Injury and Abnormalities	Fibrosis	2.63E-07
		
**Highly expressed in Inactive**		
Cell-to-Cell Signaling and Interaction	Adhesion of cells	3.38E-06
	Formation of cell-cell contacts	1.41E-03
	Formation of tight junctions	1.96E-02
	Formation of intercellular junctions	2.40E-02
Cellular development	Differentiation of epithelial cells	1.08E-03
	Maturation of epithelial cells	8.05E-03
	Differentiation of epithelial tissue	5.13E-02
Tissue Development	Morphogenesis of epithelial tissue	6.13E-03

^a^Benjamini-Hochberg adjusted *P*-values calculated in Ingenuity Pathway Analysis.

The aggregation of biological behaviors observed in the Active group, as well as some of the individual gene expression changes, represented processes associated with activated stroma or active dedifferentiation/epithelial-to-mesenchymal transition (EMT). Because extratumoral microenvironments show activation of wound response gene expression [7], and because EMT occurs normally during wound healing [26] and in cancer [23], we performed additional analyses and experiments to evaluate the hypothesis that EMT-like signatures distinguished these two clusters. EMT is a process that alters the polarity of epithelial cells and reshapes them for movement. TWIST1, TWIST2 and ZEB2 are transcription factors that bind to E-boxes within the *CDH1 *promoter, suppressing transcription and ultimately down-regulating E-cadherin protein [27,28]. Loss of E-cadherin is a key step in the dedifferentiation of epithelial cells to a mesenchymal phenotype [16]. Simultaneously, there is a loss of cell-cell contact proteins, such as claudins, and gain of mesenchymal markers, such as vimentin and S100A4 (also known as fibroblast-specific protein 1, FSP1) [29,30]. Additional file 3: Figure S1 visualizes the expression of the markers listed above and other established markers associated with a dedifferentiated phenotype, and demonstrates higher expression (relative to median) of *TWIST1, TWIST2 and ZEB1 *genes across samples in the Active Cluster. We also observed the same pattern of expression for the intermediate filament protein vimentin, the cytoskeletal protein S100A4 and the receptor tyrosine kinase DDR2. On the other hand, we found lower expression of tight junction associated proteins claudins 3, 4, 7, occludin and the calcium dependent cell-to-cell adhesion protein E-cadherin in the Active cluster.

Consistent with these observations, we identified and evaluated an epithelium-derived signature of TWIST1 activation (derived using cell-line experiments described in Methods) and the claudin-low tumor signature [23], associated with tumor cell EMT [21], for association with Active subtype. Table 3 shows Active samples were strongly associated with both the TWIST expression signature and claudin-low tumor signatures. In addition, the association with claudin-low expression was independent of whether the adjacent tumor expressed claudin-low features, and claudin-low phenotype was much more prevalent in the normal tissue (> 40%) than in the tumors (< 15%).

**Table 3:** Correlations of cancer-adjacent gene expression with biologically relevant signatures, by microenvironment subtype and tumor characteristics

		Microenvironment subtype			Tumor subtype/tumor characteristics		
**Signature**	**Number of genes^a^**	**Active** **N (%)** ***N *= 27**	**Inactive** **N (%)** ***N *= 45**	**Claudin low** **N (%)** ***N *= 9**	**Non- Claudin low** **N (%)** ***N *= 63**	**ER positive^b^** **N (%)** ***N *= 44**	**ER negative** **N (%)** ***N *= 22**

**TWIST**	831/794/664						
**positive**		25 (92.6)	7 (15.6)				
**negative**		2 (7.4)	38 (84.4)				
		*P = 5.7e-11*					
**Claudin Low**	807/754/478						
**Positive**		26 (96.3)	7 (15.6)	6 (66.7)	27 (42.9)		
**Negative**		1 (3.7)	38 (84.4)	3 (33.3)	36 (57.1)		
		*P = 3.6e-12*		*P = 0.28*			
**TGFβ**	217/207/181						
**Positive**		18 (66.7)	15 (33.3)				
**Negative**		9 (33.3)	30 (66.7)				
		*P = 7.7e-3*					
**DTF**	511/451/351						
**Positive**		16 (59.3)	16 (35.6)				
**Negative**		11 (40.7)	29 (64.4)				
		*P = 0.086*					
**ER Response**	754/700/407						
**positive**		14 (51.9)	21 (46.7)			25 (56.8)	5 (22.7)
**negative**		13 (48.1)	24 (53.3)			19 (43.2)	17 (77.3)
		*P = 0.80*				*P = 0.010*	

^a ^Correlations are presented using data for genes that mapped and had IQR > than median IQR. The number of genes for each signature are given as total number of genes in published signature/number of genes that mapped to the dataset/number of genes in dataset with IQR > median IQR. This final number of genes was used to perform association analyses.

^b ^six tumors were missing ER status.

Stroma-derived signatures were also considered in association with Active versus Inactive subtype. TGFβ signaling in the microenvironment has been implicated in tumor progression and is enriched in active wound healing, so we evaluated association of Active subtype with TGFβ signaling. Active samples were more likely to be positively correlated with the TGFβ signature, though the association was weaker than for TWIST and claudin-low signatures. We also evaluated correlation with desmoid-type fibrosis signature [4], which is associated with breast cancer progression when measured in tumors, and found no strong association between Active subtype and fibrosis. The relatively weaker association between Active subtype and each of these two stroma-derived signatures suggests that the biological characteristics of Active versus Inactive are more similar to the cellular dedifferentiation captured in the TWIST and claudin-low signatures.

**Figure 2:**
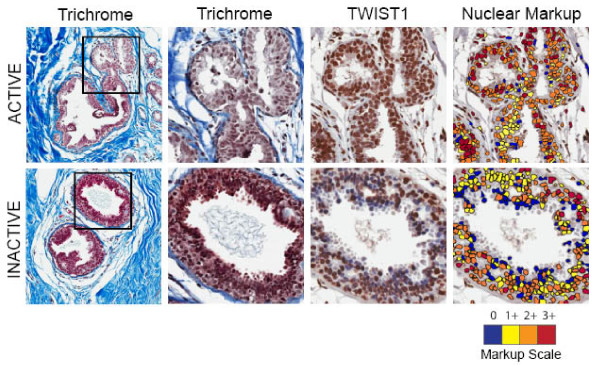
**Active phenotype is associated with increased density of TWIST positive cells**. Representative Active and Inactive tissues are shown. Two trichrome images are shown (different magnifications) and the TWIST1-DAB stained section illustrates that despite a greater density of epithelial cells in the Inactive patient, there is a lower density of TWIST staining and the intensity of staining (nuclear markup image) is reduced relative to Active patients. The nuclear markup scale ranges from blue (no staining) to red (3+ intensity).

To identify protein level overexpression of TWIST and to identify the source of the TWIST protein in breast cancer samples, we performed immunohistochemistry along with Masson's trichrome in clinical specimens. Aperio scanned images analyzed by ImageScope software demonstrated that the density of TWIST positive cells was higher in both epithelium and stroma of Active patients (73% increase in density of TWIST positive cells in epithelium, 44% increase in density of TWIST positive cells in stroma relative to Inactive group). Two representative ductal specimens, one each from Active and Inactive patients, depicting Masson's Trichrome, the TWIST-stained samples, and Aperio ImageScope scoring for nuclear markup/quantitative scoring are shown in Figure 2. The images show that even though the pictured Inactive specimen had more epithelial cells, there are far fewer red and orange (3+ and 2+, respectively) cells relative to what is seen in the Active sample, and there are a larger number of yellow and red (negative or 1+) cells. These images illustrate the trend observed across the entire IHC sample set: the density of intense TWIST staining was higher among Active patients.

In tumors, estrogen response gene expression strongly distinguishes two groups of patients and often drives progression [31]. To ensure that the subtypes were not simply measuring estrogen responsiveness of the tumors and, thereby, acting as a surrogate for ER status of the tumors, we evaluated a published estrogen response signature [24]. Consistent with previous reports that ER positive tumors are more likely to show high estrogen response gene expression in adjacent epithelium, the estrogen responsiveness signature was more likely to be positive in tissue adjacent to ER positive breast cancers. However, the Active and Inactive subtypes were independent of the estrogen response gene set, with no association between Active microenvironment and estrogen response gene expression. In other words, the Active patients are not defined by estrogen responsiveness.

### Prevalence of subtypes as a function of distance from tumor

Spatial variation of the Active signature across a patient's tissue is an important consideration if the signature represents a candidate biomarker of prognosis among ER positives. For our unsupervised clustering in Figure 1, samples were taken peritumorally, but tissue was not sampled distant from the tumor and precise distances were not recorded. Thus, in a subset of samples, we specifically evaluated both peritumoral and remote tissue to identify whether two locations from the same patient were concordant (Additional file 4: Table S3). Interestingly, 60% of patients had concordant normal tissue signatures at both locations, while 40% of patients had Active signature in one location and not in the other, (that is, differences existed between peritumoral and remote signatures). This suggests that distance to tumor may be an important source of intraindividual variation in expression of this phenotype.

## Discussion

Pathways and processes that are part of normal homeostasis, such as wound healing and TGF-β signaling, become tumor promoting in the presence of initiated cells [32,33]. While this has been proven experimentally, the role of these processes in predicting human cancer outcomes has been less well studied in observational contexts. We previously observed that wound healing signatures can be detected in more than 90% of tumor adjacent tissues [7], and in the current study, we demonstrate that extratumoral wound response can be of two types: Active and Inactive. Active cancer-adjacent tissue shows features of active EMT or cellular dedifferentiation and was associated with poor survival. Inactive extratumoral tissue maintains higher expression of cellular adhesion genes and shows lower expression levels of EMT-related transcription factors. Our preliminary estimate of the hazard ratio (HR) associated with Active (compared to Inactive) microenvironment was approximately 2.5, which is substantial considering that established genomic phenotypes such as p53 mutation status [34] and Cyclin E overexpression [35] have HRs near two. Acknowledging the limitations of this study, which included heterogeneously treated patients of various stages, the magnitude of this preliminary effect estimate suggests that the association merits further investigation for clinical relevance and lends credence to the influence of tumor microenvironment on breast cancer outcome.

The clinical relevance of our gene expression subtypes appeared, in our study, to be restricted to ER-positive patients. It is common for tumor-derived gene expression signatures to have prognostic value only among ER-positive tumors, which may be attributable to relatively uniform, poor survival among ER-negative patients. A recent paper simultaneously reviewed multiple gene expression signatures and showed that very few tumor signatures had value in predicting ER-negative breast cancer survival [25]. However, it may also be the case that there are specific biological interactions between ER-positive tumors and their microenvironments. For example, TGF-β is a well-established paracrine mediator of breast cancer aggressiveness [36,37], and consistent with our findings, TGF-β signatures are prognostic solely among ER-positive cancers [38]. This example is particularly relevant given that Active microenvironments are correlated with a TGF-β signature.

Increased TGF-β signaling along with other biological characteristics of the Active microenvironment subtype suggested dedifferentiation or EMT-like gene expression, which may seem surprising given that EMT is a developmental program. However, EMT is known to be activated in normal, adult tissues undergoing wound healing (referred to as type 2 EMT, in contrast to type 1 EMT that occurs during development) [26]. In tumors and cancer cells, EMT-associated expression has been observed [39-41] (referred to as type 3 EMT), and has recently been linked with the claudin-low subtype and with resistance to therapy [21,23,26,42]. In our study, we observed that claudin-low gene expression features are more common (more than twice as likely to occur) in cancer-adjacent tissues than in breast tumors, and occur independent of whether the adjacent tumor is claudin-low. In fact, claudin-low gene expression in the adjacent tissue appears to be independent of tumor subtype altogether. This suggests that the Active signature may be an endogenous response, dependent upon patient biology and not tumor biology. It also appears that the signature can occur early in disease pathogenesis, being present in some DCIS patients in the current study.

A recent study has suggested that claudin-low features may also occur (though much less frequently) in normal tissues from non-diseased women [43]. In that study, claudin-low features were associated with high risk of breast cancer. Future studies should assess whether the prevalence of this subtype differs according to disease progression (DCIS versus Invasive), or other patient characteristics, such as age, smoking history or race. In one study of esophageal cancers, it has been hypothesized that EMT could be artificially induced in surgery [44], consistent with the idea that EMT is induced during wounding. However, tumors displaying this signature were exposed to ischemic conditions for at least four hours. In collections at UNC, more than 95% of specimens are snap frozen within two hours of devascularization, with the majority snap frozen in under an hour. Thus induction of ischemic conditions is unlikely to explain our observation of EMT-like gene expression.

In support of an exogenous or tumor-specific induction of EMT-like gene expression, EMT marker expression has been previously reported as a highly localized response to cancer progression. Trujillo *et al. *[45] observed high expression of SNAIL1 and TGFβ in the breast epithelium adjacent to tumors. Interestingly, across the five patients evaluated in that study, these markers were most highly expressed in the peritumoral region (< 1 cm) and expression was much lower at a distance of 5 cm from the tumor margin. In our analyses, many pairs of peritumoral and remote tissue had pervasive expression of an EMT-like signature, both near (< 2 cm) and remote from the tumor (> 2 cm). Differences between the number of markers (hundreds of genes versus few markers) and differences in the sensitivity of RNA-based and protein-based analyses may account for differences between our study and the report by Trujillo *et al. *However, future work should continue to evaluate the role of distance from tumor in modifying these biological processes. If there is a wide geographic range of the tumor-effects on adjacent tissue, this has potential implications for surgery strategies. One might speculate that patients for whom a widespread gene expression alteration is present might benefit from mastectomy. If a promoting wound healing signature or Active signature persists in the lumpectomy bed, it raises the question of whether this could account for the higher rate of recurrences in this region after breast conserving therapy. If this hypothesis is to be advanced, it will be important to evaluate whether Active/Inactive signatures persists after breast conserving therapy.

Other studies of stroma-derived signatures have supported both exogenous and endogenous origins for stromal response [46]. Whether the signature is tumor-dependent or a host factor varies signature by signature. In our findings, estrogen response signatures in extratumoral tissue were correlated with tumor ER status. However, neither the tumor ER status nor the estrogen responsiveness of the adjacent tissue were correlated with the extratumoral subtypes we identified, suggesting that the extratumoral phenotypes are not driven by hormonal exposures or endogenous hormone response. These results are important because breast tumor biology is so strongly driven by estrogen response and because previous reports have shown that estrogen positive tumor features are reflected in the adjacent normal tissue of these patients [13]. However, while our Active and Inactive subtypes are independent of hormone receptor status of the adjacent tumor, we did recapitulate the finding of Graham *et al. *[13] that extratumoral estrogen responsiveness mirrors the ER expression phenotype of the adjacent tissue, despite the fact that we did not micro-dissect the epithelium from the stroma. Thus, we are able to report that even signatures such as estrogen responsiveness are detectable in whole (not micro-dissected) tissue. The use of whole tissue facilitates microarray analysis of a much larger number of samples (more than 90 in the current study) facilitated the assessment of clinical associations and inter-and intra-individual variation that would not have been possible with smaller sample sizes that have been used in micro-dissection studies [1,13].

The higher prevalence of Active subtype in our study of women with DCIS and cancer (compared to the study of non-diseased high risk patients) suggests that either the signature is associated with risk or that initial activation of the signature requires the presence of tumor or another early benign lesion [7]. Further research to determine temporality and distinguish risk and response is needed. It will also be important to determine whether there are some extratumoral signatures that are dictated by tumor characteristics, to fully explore the role of the extratumoral tissue in disease progression. For example, grade is known to induce widespread stromal alterations, but the specific molecular signatures associated with high grade tumors are not well studied. Likewise, extratumoral microenvironments that are common to basal-like breast cancers and could account for higher risk of loco-regional recurrence could provide novel insights about the biology of basal-like breast cancer progression.

## Conclusions

We have explored novel subtypes of extratumoral microenvironment that appear to be independent of breast cancer subtype and that may have prognostic value. We have shown that these subtypes have distinct biological characteristics that may determine their clinical impact. While the extratumoral subtypes we identified were not dependent on tumor characteristics, we have confirmed the findings of others that some extratumoral characteristics vary according to the ER-status of the adjacent tumor. These results demonstrate that studying the cancer-adjacent tissue can provide novel biological and clinically-relevant insights about breast cancer progression.

## Abbreviations

DCIS: ductal carcinoma *in situ*; DTF: desmoid-type fibrosis; EMT: epithelial-to-mesenchymal transition; ER: estrogen receptor; FDR: false discovery rate; HMLE: human mammary epithelial cells; HR: hazard ratio; IPA: Ingenuity Pathway Analysis; IQR: interquartile range; RMF: reduction mammoplasty fibroblast; SAM: Significance Analysis of Microarrays; TGF-β: transforming growth factor beta.

## Competing interests

The authors declare that they have no competing interests.

## Authors' contributions

ERP performed microarray and clinical data analyses, analyzed images for staining, and drafted the manuscript. PCH helped to coordinate the sample processing and contributed to drafting the manuscript. JRP performed microarray analysis and contributed to drafting the manuscript. JR performed RNA extraction from tissues, labeled the RNA and performed hybridizations to microarrays. RAL helped design the study and interpret the results. LAC provided access to clinical data and contributed to data interpretation. SAM participated in the selection and interpretation of EMT-associated markers and helped to draft the manuscript. KDA and MAT conceived of the study, participated in its design and coordination, and helped to draft the manuscript. All authors read and approved the final manuscript.

## Supplementary Material

Additional file 1 **Table S1. Complete gene list used for unsupervised clustering and ingenuity pathway analysis**. Each gene in Figure 1A is presented in this table showing average median-centered log2(R/G) in Active and Inactive groups. Fold change is calculated from the ratio of columns C and D. Genes highlighted in red are those in the gene cluster marked in orange in Figure 1A and genes highlighted in green are the gene cluster marked in grey in Figure 1A. These gene clusters were used to perform the IPA analyses presented in Table 1 and Table S2. Click here for file

Additional file 2 **Table S2. Ingenuity Pathway Analysis of Molecular and Cellular Functions associated with gene clusters in **Figure 1A. Gene categories, functions, function annotation, Benjamini-Hochberg *P*-value and lists of molecules detected per category are shown for each of the two clusters (orange and grey) identified in Figure 1 and enumerated in Table S1. Click here for file

Additional file 3 **Figure S1. Identification of EMT markers in extratumoral microenvironment subtypes in Active and Inactive patients**. EMT-associated genes selected from the literature are visualized across the two sample groups from Figure 1. Click here for file

Additional file 4 **Table S3. Concordance of extratumoral subtypes in paired tissues from the same patient**. At least two patient samples were used for microarray analysis and Active versus Inactive subtype was evaluated in each. Samples include specimens from the University of North Carolina at Chapel Hill Normal Breast Study and samples collected in the NCI-funded Polish Women's Breast Cancer Study. Click here for file
