# Supplementary figures and images for: Gene expression in extratumoral microenvironment predicts clinical outcome in breast cancer patients
Source: Breast Cancer Res. 2012 Mar 19;14(2):R51. doi: 10.1186/bcr3152 (PMC3446385; doi:10.1186/bcr3152)

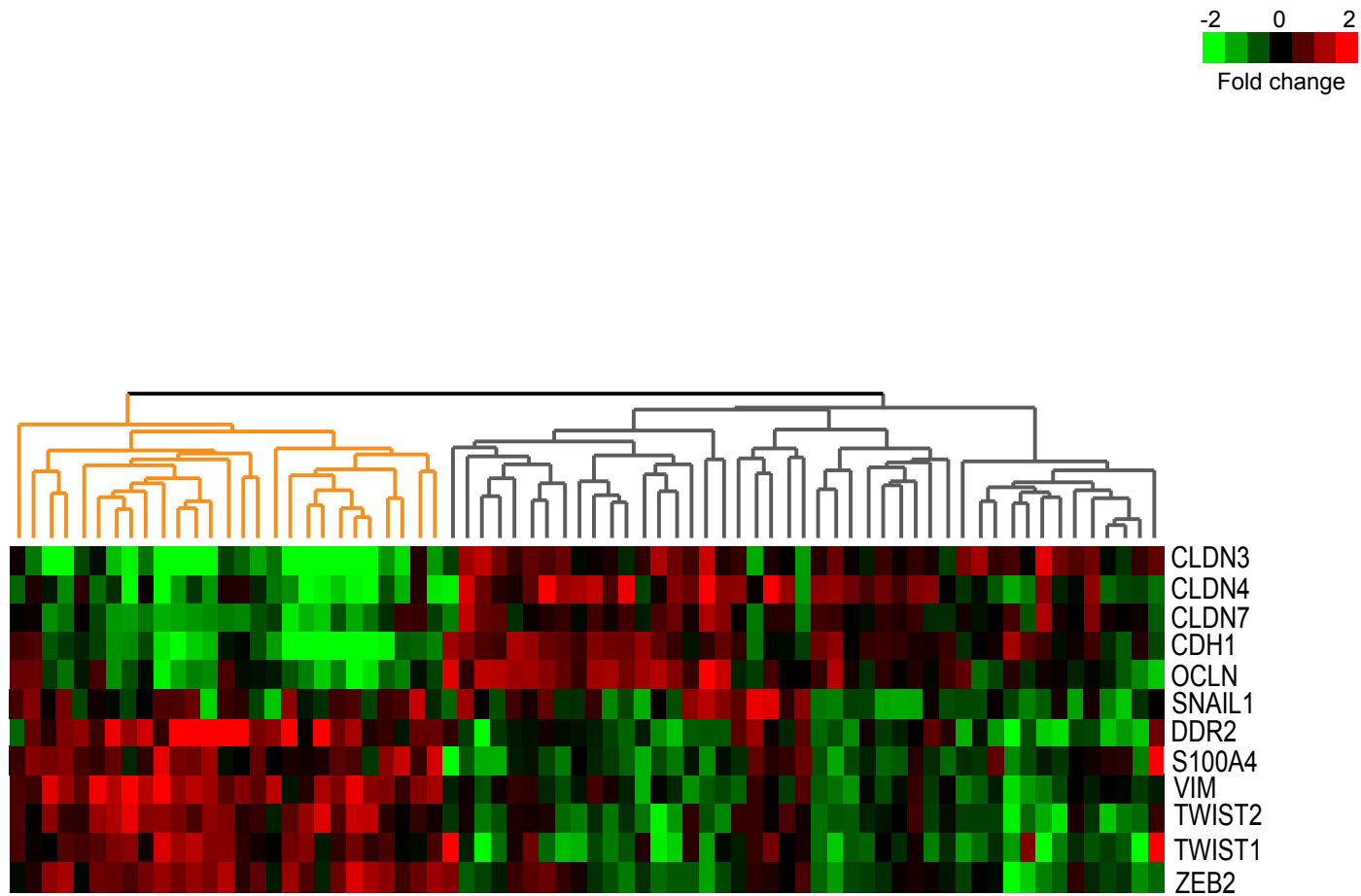

Supplement: Additional file 3 — Figure S1. Identification of EMT markers in extratumoral microenvironment subtypes in Active and Inactive patients. EMT-associated genes selected from the literature are visualized across the two sample groups from Figure 1. [file bcr3152-S3.PDF]
