# Supplementary material for: Gene expression in extratumoral microenvironment predicts clinical outcome in breast cancer patients
Source: Breast Cancer Res. 2012 Mar 19;14(2):R51. doi: 10.1186/bcr3152 (PMC3446385; doi:10.1186/bcr3152)
Supplement: Additional file 4 — Table S3. Concordance of extratumoral subtypes in paired tissues from the same patient. At least two patient samples were used for microarray analysis and Active versus Inactive subtype was evaluated in each. Samples include specimens from the University of North Carolina at Chapel Hill Normal Breast Study and samples collected in the NCI-funded Polish Women's Breast Cancer Study. [file bcr3152-S4.DOCX]

**Supplemental Table 3. Concordance of extratumoral subtype in paired tissues from the same patient.**

| **Patient ID** | **Distance to Tumor** | **Active/Inactive** | **Concordant?** |
| --- | --- | --- | --- |
| NBS10027 | <1 cm | Active | No |
|  | 4+ cm | Inactive |  |
| NBS10038 | <1 cm | Active | No |
|  | <1 cm | Active |  |
|  | 2-<4 cm | Inactive |  |
|  | 4+ cm | Inactive |  |
| NBS10047 | <1 cm | Inactive | Yes |
|  | <1 cm | Inactive |  |
|  | <1 cm | Inactive |  |
|  | 4+ cm | Inactive |  |
| NBS10051 | <1 cm | Inactive | No |
|  | 4+ cm | Active |  |
| NBS10080 | <1 cm | Active | Yes |
|  | 4+ cm | Active |  |
| NCI10050 | <2 cm | Inactive | Yes |
|  | 2+ cm | Inactive |  |
| NCI10081 | <2 cm | Active | Yes |
|  | 2+ cm | Active |  |
| NCI10238 | <2 cm | Active | No |
|  | 2+ cm | Inactive |  |
| NCI10261 | <2 cm | Active | Yes |
|  | 2+ cm | Active |  |
| NCI10263 | <2 cm | Inactive | Yes |
|  | 2+ cm | Inactive |  |
